# *Nocardia otitidiscaviarum* causing pulmonary nocardiosis: a case report and its review of the literature

**DOI:** 10.1099/acmi.0.000530.v5

**Published:** 2024-02-16

**Authors:** Saumya Srivastava, Arghadip Samaddar, Salman Khan, Vibhor Tak, Gopal Krishna Bohra, Deepak Sharma, Arnab Ghosh, Vijaya Lakshmi Nag

**Affiliations:** ^1^​ Department of Microbiology, All India Institute of Medical Sciences, Jodhpur, India; ^2^​ Department of Microbiology, All India Institute of Medical Sciences, Delhi, India; ^3^​ Department of General Medicine, All India Institute of Medical Sciences, Jodhpur, India; ^4^​ Department of Microbiology, Post Graduate Institute of Medical Education and Research, Chandigarh, India

**Keywords:** pulmonary nocardiosis, pleural nocardiosis, *Nocardia otitidiscaviarum*, MALDI TOF MS, Ziehl-Neelsen, Ziehl-Neelsen staining

## Abstract

**Background.:**

Infections caused by *Nocardia* spp. can occur in immunocompromised as well as immunocompetent individuals. Although nocardiosis is rare, it is being increasingly recognized owing to the rise in occurrence rate over the years. The documentation of pleural involvement in nocardiosis is rare in India.

**Case.:**

We report a case of pulmonary nocardiosis in an immunocompromised individual caused by *Nocardia otitidiscaviarum*.

**Discussion.:**

Pulmonary nocardiosis caused by *Nocardia otitidiscaviarum* may go unnoticed without clinical suspicion. Correct and timely identification is the key to proper patient management.

**Conclusion.:**

Coordination between clinicians and microbiologists is necessary for early diagnosis and appropriate management of nocardiosis.

## Introduction

*Nocardia* species are facultative intracellular pathogens. Their ability to grow in macrophages and polymorphonuclear leukocytes is undoubtedly important for their ability to produce infection [1]. Infection caused by *Nocardia* spp. is known as nocardiosis. Disease manifestations may range from localized skin infections in immunocompetent patients to serious haematogenous spread to various organs, including lungs and the central nervous system in immunocompromised individuals [2]. Out of more than 100 different species known under the genus *Nocardia,* more than 40 are considered clinically relevant [3]. The most common species are from the *N. asteroides* complex (*N. asteroides sensu stricto*, *N. farcinica, N. nova* and *N. abscessus*). Other medically important species are *N. brasiliensis, N. otitidiscaviarum, N. africana, N. brevicatena* complex, *N. carnea, N. paucivorans, N. pseudobrasiliensis, N. transvalensis* and *N. veterana*).

*Nocardia otitidiscaviarum* (*N. otitidiscaviarum*) is an opportunistic pathogen causing localized and/or disseminated infection in immunocompromised and immunocompetent individuals. It was first isolated in 1924 from guinea pigs. *N. otitidiscaviarum* (formerly *N. caviae*) has been isolated from the soil throughout the world. Compared to other *Nocardia* species, it is less commonly implicated in disease aetiology. The distribution and clinical relevance of other rarely isolated species of *Nocardia* are less clear, although molecular and mass spectrometry methods for identification may change this in the future [4–6].

## Case presentation

**Fig. 1.**
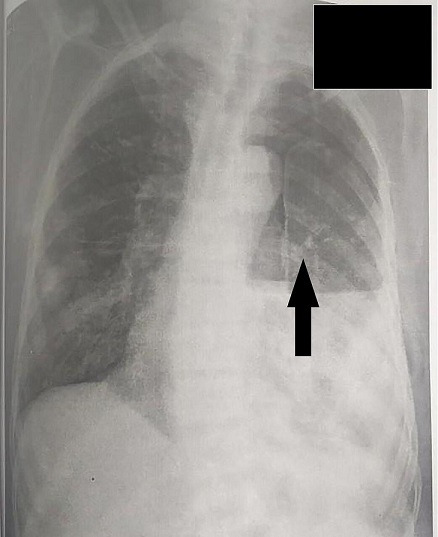
Increased broncho-vascular marking. (Marked with arrow).

A 55-year-old male presented to the emergency department on 10th of January 2019 with complaints of breathlessness for seven to eight months and increased shortness of breath for one month. He had a history of chest pain at rest which increased on coughing. He also complained of episodes of syncope occasionally. He also gave a history of on-and-off fever for one month. He had a high-grade fever for 2 days one month back, which was without chills and rigors. There was no history of hemoptysis and palpitations. The patient was a known case of hypertension, for which he was on medication for the last seven months. He was a known case of ischemic heart disease with old inferior wall myocardial infarction two years back. He was taking anti-platelets, beta blockers and nitrates for the last seven months. He was also diagnosed with chronic kidney disease seven months back and was on hemodialysis for the last two months. There was no history of diabetes mellitus. There was a history of pulmonary tuberculosis 20 years back for which he had taken anti-tubercular treatment for two years. There was a history of hospital admission due to pneumonia one month back. On examination, the patient had a thin build. His pulse rate was 90 per minute, blood pressure 90/50 mm of Hg, respiratory rate 22 per minute and body temperature 98.2 °F. The patient had severe dyspnoea, bilateral crepitations with decreased air entry, with left-sided pneumothorax, for which an inter-costal drainage tube was placed. On the electrocardiogram, persistent ST elevation with Q wave was present. Echocardiography showed the left ventricle and left atrium were dilated, ejection fraction was 10–15 %. X-ray showed increased bronchovascular markings. (Fig. 1 marked with arrow). The patient’s haemogram revealed Hb: 8.3 (g dl^−1^), WBC: 2.32(103 /ul), and RBC: 3.94(10^6^ /ul). His renal function tests (RFT) were deranged with serum urea: 137 mg dl^−1^, creatinine: 1.64 mg dl^−1^, sodium: 146 mmol l^−1^, potassium: 4.18 mmol l^−1^, and chloride-18mmol l^−1^. His liver function tests (LFT) were partly deranged with SGPT: 14 U l^−1^, SGOT: 18 U l^−1^, total bilirubin: 0.65 mg d^l−1^, direct bilirubin: 0.22 mg d^l−1^, indirect bilirubin: 0.43 mg dl^−1^, total protein: 5.55 g dl^−1^, albumin: 2.19 g dl^−1^, globulin: 3.36 g dl^−1^, and ALP: 239 U l^−1^. He was started empirically on piperacillin-tazobactam 2.25 g IV eight hourly, Meropenem 1 g IV BD (bis in die or twice daily) and Amikacin 300 mg IV BD.

**Fig. 2.**
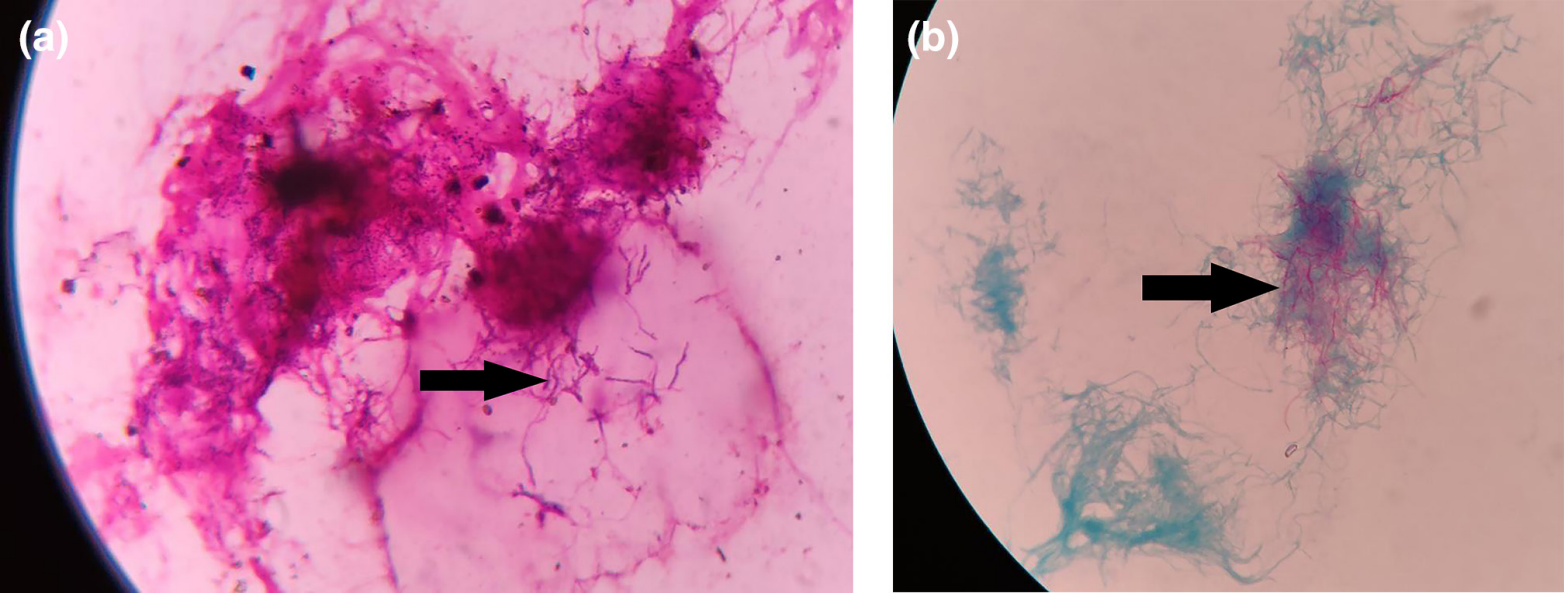
(a) Gram stain of pleural fluid showing Gram-positive filamentous bacteria (marked with arrow). (b) Modified Ziehl-Neelsen staining revealed acid-fast filamentous bacteria (marked with arrow).

**Fig. 3.**
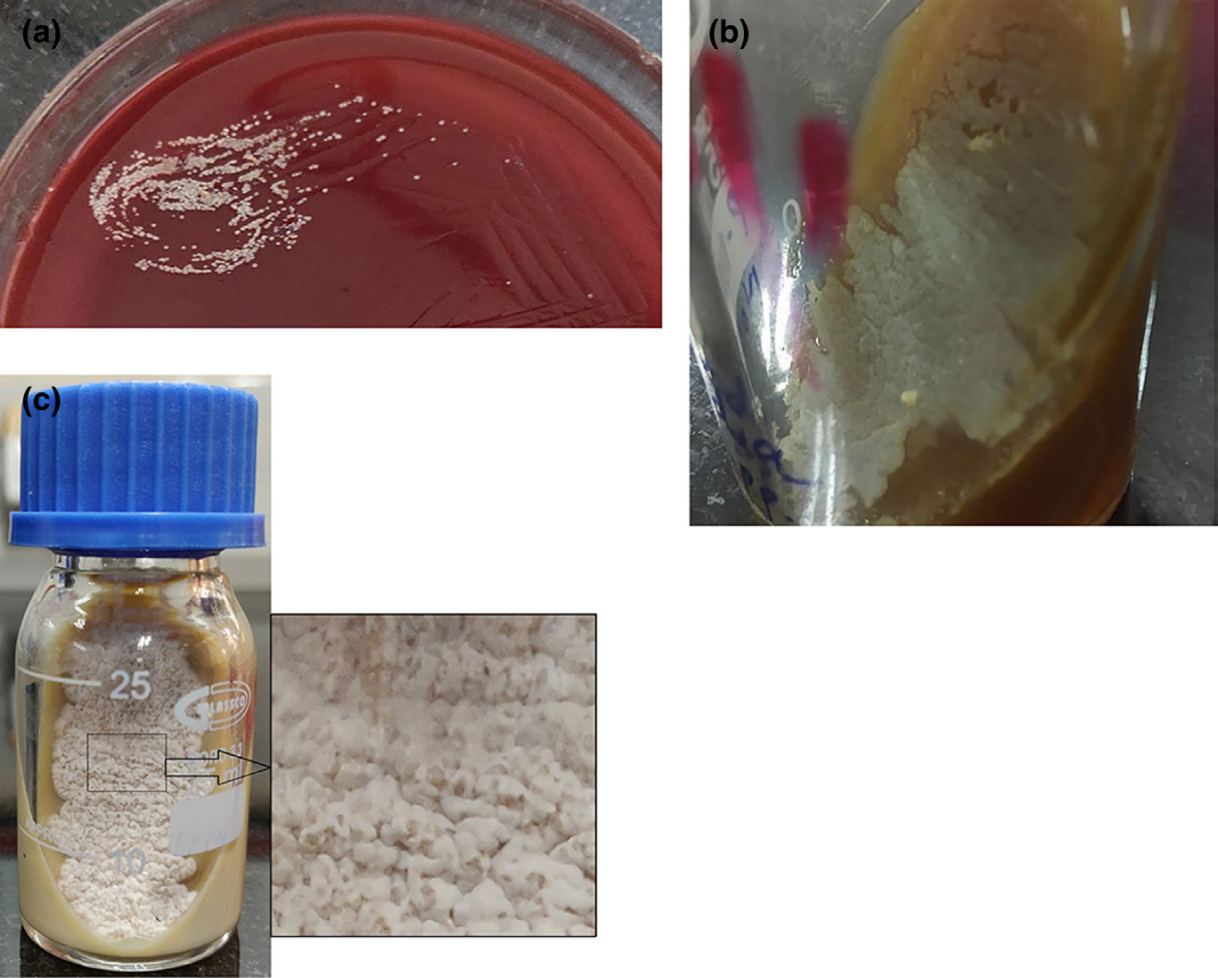
(a) On Blood agar non-haemolytic, chalky-white dry colonies after 48 h of incubation. (b) On Blood agar slope non-haemolytic, dry chalky-white dry colonies after 48 h of incubation. (c) On Lowenstein–Jensen media showing dry whitish colonies, which on further incubation showed the typical raised, chalky-white dry colonies after 48 h of incubation.

Pleural fluid was sent to the microbiology laboratory on 10 January 2019 for Gram stain, aerobic bacterial culture and sensitivity and Ziehl-Neelsen (ZN) staining for acid-fast bacilli. A Gram-stained smear of pleural fluid showed the presence of occasional pus cells and Gram-positive thin, beaded, branching, filamentous bacteria (Fig. 2a marked with arrow). Modified ZN staining using 1 % sulphuric acid showed acid-fast branching filaments suggestive of *Nocardia* species (Fig. 2b marked with arrow). On blood agar (Fig. 3a, b), the colonies were non-haemolytic dry chalky-white and dry and whitish on Lowenstein–Jensen medium, which on further incubation showed the typical raised, chalky-white dry colonies after 48 h (Fig. 3c). Gram-stained smear from the culture also showed Gram-positive filamentous bacteria and modified ZN staining with 1 % sulphuric acid revealed acid-fast filamentous bacteria. The isolate was confirmed and speciated as *Nocardia otitidiscaviarum* by VITEK MS (bioMérieux, France) with a confidence value of 99.9 %. VITEK MS works on the principle of matrix-assisted laser desorption ionization-time of flight mass spectrometry (MALDI-TOF-MS).

Antimicrobial therapy was changed to a combination of meropenem 1 gm IV eight hourly and trimethoprim/sulfamethoxazole 1600/320 mg twice daily after confirmation of *Nocardia* by the Microbiology department on 12 January 2019. The patient improved clinically on modified treatment. The patient remained afebrile, with significant improvement in chest pain and breathlessness for the next 2 weeks. After 2 weeks, the patient developed upper gastrointestinal bleed, with thrombocytopenia (platelet count 20 000 per microlitre) with suspected septic shock but blood cultures showed no growth. Although the patient was being managed with optimal fluid, vasoactive and inotrope support, he further deteriorated and had altered sensorium with a platelet count of 1000 per microlitre. Finally, even after the best possible efforts including cardio-pulmonary resuscitation, the patient succumbed to his illness on 26 January 2019.

## Discussion

*Nocardia otitidiscaviarum* causes local or disseminated opportunistic infections in immunocompetent as well as immunocompromised patients [7, 8]. The epidemiology of nocardiosis is evolving. With better identification methods, *Nocardia* species are being increasingly identified in human infections [8]. Though nocardiosis prevalence in India is unclear, a few studies indicate that it varies from 1.37–1.9 % [9–12]. The lung is the most common organ primarily affected, accounting for 60–70 % of all human nocardiosis [13]. Mortality due to nocardiosis ranges from 40–80 % depending on the immune status of the patient and intervention therapy [13–17]. *Nocardia* species can cause disseminated, cutaneous and rarely catheter-related infections and healthcare-associated diseases [4, 9–18].

Li *et al*. performed a retrospective analysis of 24 patients of both autoimmune disease and pulmonary nocardiosis, which revealed the involvement of only lung in 11 cases (45.8 %); lung and skin in seven cases (29.2 %); lung, pleura and brain in two cases (8.3 %); lung and pleura in only one case as this is very rare (4.2 %); lung, skin and brain in one (4.2 %) case; lung, pleura and skin in one case (4.2 %); lung, pleura, brain and skin in one (4.2 %) case [19]. We have performed a literature review on PubMed and Google scholar using the search term pulmonary nocardiosis, which yielded the published articles enlisted in Table 1.

**Table 1.** Review of characteristics and management of nocardiosis

Author	Year	Age/ gender	Site/ sample	Treatment	Risk factors	Time gap between onset of symptoms and definitive therapy	Patient outcome	Ref.
Fu *et al*.	2023	73 /M	BAL, skin lesions	TMP-SMX, amikacin and levofloxacin	COPD	na	Expired	[27]
Barry *et al*.	2022	37 /F	Brain, skin lesions	TMP-SMX and imipenem-cilastatin for 3 weeks then it became resistant to these drugs. Further, the susceptibility was changed to amikacin, linezolid, moxifloxacin, and doxycycline.	Carcinoma breast, steroid therapy, gardening.	1 month	Expired	[20]
Kullab *et al*.	2022	36 /F	Lung, skin, and brain abscess	Amikacin, linezolid, and ceftriaxone. Then moxifloxacin was added after 5 weeks.	Carcinoma breast, chemo-therapy.	5 weeks	Improved	[28]
Albuni *et al*.	2022	47 /M	Right knee joint- septic arthritis	TMP-SMX 240 twice daily (I.V.), moxifloxacin 400 mg, and prednisolone.	Nephrotic syndrome, steroid therapy	na	Improved	[29]
Das *et al*.	2022	42 /M	Brain	I/V TMP-SMX (5 mg/kg/day 8 hourly) and imipenem (500 mg 6 hourly) were initiated. After 4 weeks, the patient was continued on oral TMP-SMX only.	Steroid therapy, old tuberculosis	2 m	Improved	[30]
Kudru *et al*.	2021	48 patients, 33 males and 15 females	BAL and Sputum	TMP-SMX, carbapenem, ceftriaxone, and amikacin.	Renal transplant, steroid use, HIV, chemo-therapy, DM, CKD, COPD, asthma, tuberculosis	na	Six patients expired.	[21]
Toyokawa *et al*.	2021	Not mentioned	Respiratory specimens, skin and soft tissue, blood, deep abscess, pleural effusion, ascitic fluid, synovial fluid (153 isolates)	TMP-SMX, amikacin, tobramycin, ceftriaxone, imipenem, minocycline, linezolid, ciprofloxacin, moxifloxacin, clarithromycin, cefotaxime, meropenem, tigecycline and arbekacin.	na	na	na	[31]
Parengal *et al*.	2021	29 /F	Sputum, BAL, skin pustules	TMP-SMX (MIC 1 µg ml^−1^), amikacin (MIC 2 µg ml^−1^), ciprofloxacin (MIC 0.5 µg ml^−1^), moxifloxacin (MIC 0.25 µg ml^−1^) and linezolid (MIC 0.094 µg ml^−1^). It was resistant to amoxicillin-clavulanate (MIC 32 µg ml^−1^), ceftriaxone (MIC32 μg ml^−1^) and clarithromycin (MIC 12 µg ml^−1^).	SLE with multi-organ involvement	na	Improved	[26]
Sah *et al*.	2020	61 /M	Subcutaneous abscess and pulmonary infection	Meropenem and amikacin in addition to TMP-SMX (6 months)	Nephrotic syndrome, steroid therapy	na	Improved	[32]
Liu *et al*.	2017	58 /M	Lung	Amikacin, imipenem and TMP-SMX.	Hepatitis B carrier, respiratory disease	13 days	Expired	[33]
Savitha *et al*.	2017	65 /M	Middle zone of both lungs	Oral TMP-SMX (double strength BD) for 12 weeks with dose reduction after 4 weeks.	Not present	na	Improved	[34]
Deepa *et al*.	2016	14 /F	Lung- pleural Effusion	Gentamicin, ciprofloxacin, TMP-SMX, amikacin, tetracycline, imipenem.	Rheumatic heart disease	2 weeks	Expired	[23]
Bagali *et al*.	2016	45 /M	Pleural Nocardiosis	Patient died before the initiation of specific treatment.	Rheumatic heart disease	na	Expired	[35]
Shahapur *et al*.	2014	60 /M	Ulcer on right thigh (Inguinal lymph nodes)	TMP-SMX for 2 weeks (no improvement), amikacin, minocycline and linezolid.	Thorn prick, COPD	na	Improved	[36]
Chi *et al*.	2013	71 /M	Granulation tissue on left lower leg	Penicillin, ampicillin, imipenem, TMP-SMX, gentamicin, ciprofloxacin, amikacin, and antifungals.	Not present	28 years	Improved	[37]
Pelaez *et al*.	2009	85 /M	Brain and lung	Penicillin, ampicillin, imipenem and TMP-SMX, gentamicin, ciprofloxacin and amikacin.	COPD, steroid therapy	na	Expired	[38]
Karanam *et al*.	2008	23 /F 17 /M	Lung- Pleural effusion BAL fluid	Amikacin, imipenem and TMP-SMX cefepime and ciprofloxacin.	Renal transplant, steroid therapy	na	Expired	[39]

BAL, Broncho-alveolar Lavage; BD, Bis in DieCKD, Chronic Kidney Disease; COPD, Chronic Obstructive Pulmonary Disease; DM, Diabetes mellitus; HIV, Human Immunodeficiency Virus; MIC, Minimum Inhibitory Concentration; na, Not available; Ref, Reference; SLE, Systemic Lupus Erythematosus; TMP-SMX, trimethoprim/sulfamethoxazole.

Specific points regarding sites/samples involved in *Nocardia* infection, age and gender of patients, underlying risk factors, management, and clinical outcomes of patients in previous studies have also been depicted in Table 1. Several risk factors have been implicated in nocardial infections, e.g. respiratory diseases, cancer, chemotherapy, prolonged steroid use, kidney disorders, organ transplant, history of tuberculosis, heart diseases and trauma, etc. The patient in the present study also had multiple underlying risk factors like history of pulmonary tuberculosis, chronic kidney disease and heart disease. Apart from pulmonary infections as in our case, *Nocardia* infections may involve various other sites including skin and soft tissue, brain tissue and joints. Our patient was being managed with a combination of meropenem 1 gm IV eight hourly and trimethoprim/sulfamethoxazole 1600/320 mg twice daily. Different antimicrobials mostly in combinations have been used for the management of nocardiosis patients, common ones being trimethoprim/sulfamethoxazole, amikacin, carbapenems, linezolid, fluoroquinolones mainly moxifloxacin. The clinical outcome of patients depends on multiple factors. Availability of early diagnosis and antibiotic susceptibility and early initiation of definitive therapy are associated with positive outcomes, whereas high clinical severity, secondary infections, presence of risk factors and comorbidities, and delay in diagnosis and treatment are associated with negative outcomes [20–23].

Nocardiosis treatment should incorporate the use of antibiotics, incision and drainage, surgical excision of the lesion and immunity-boosting protocols [24, 25]. Sulfonamide-based antibiotics, e.g. trimethoprim/sulfamethoxazole for 3–12 months are the first choice drugs but other drugs like minocycline, doxycycline, amoxicillin-clavulanate, carbapenem, amikacin, cefuroxime, ceftriaxone, clarithromycin, ofloxacin, linezolid and inhaled aminoglycoside could be used in the case of sulfonamide allergy [8, 9]. Infectious Diseases Community of Practice of the American Society of Transplantation has published treatment guidelines for nocardiosis along with interspecies variabilities of various antimicrobials and also strongly recommends antimicrobial susceptibility testing for the effective management of such patients. Most *Nocardia* spp. were reported to be susceptible to trimethoprim/sulfamethoxazole and linezolid but resistant to clarithromycin/azithromycin. Resistance to imipenem is seen in *N. braziliensis* and *N. otitidiscaviarum*; to ceftriaxone in *N. farcinica* and *N. otitidiscaviarum*; to ciprofloxacin in *N. abscessus*, *N. nova* complex, *N. braziliensis* and *N. cyriacigeorgica*; amoxicillin-clavulanate in *N. nova* complex and *N. otitidiscaviarum*; moxifloxacin in *N. abscessus* and *N. nova* complex [22].

Infections due to *N. otitidiscaviarum* are infrequently reported. It accounted for just 0.3–2.9 % of all nocardial infections in some studies [26]. The present case is significant as it describes rare pulmonary involvement due to *N. otitidiscaviarum* infection.

### Conclusion

Pleural nocardiosis being a serious life-threatening infection in immunocompromised hosts can lead to significant morbidity and mortality. It is often missed at times due to a lack of proper identification. Thus, it is necessary to consider nocardiosis as one of the differential diagnoses in tropical countries for the proper management of the patients. It would be helpful if modified ZN stain is done for all the respiratory samples received in the microbiology laboratory so that *Nocardia* infection is not missed. Thus, early diagnosis of nocardiosis is extremely important, more so in immunocompromised patients so that appropriate treatment is initiated promptly to reduce the associated morbidity and mortality.
